# Sex and gender differences in migraines: a narrative review

**DOI:** 10.1007/s10072-022-06178-6

**Published:** 2022-06-08

**Authors:** Maria Francesca Rossi, Antonio Tumminello, Matteo Marconi, Maria Rosaria Gualano, Paolo Emilio Santoro, Walter Malorni, Umberto Moscato

**Affiliations:** 1 Department of Life Sciences and Public Health, Section of Occupational Health, Università Cattolica del Sacro Cuore, Largo Francesco Vito 1, 00168 Rome, Italy; 2 Center for Gender-Specific Medicine, Istituto Superiore Di Sanità, Rome, Italy; 3 Department of Public Health Sciences and Paediatrics, University of Torino, 10124 Torino, Italy; 4 Department of Woman and Child Health and Public Health, Fondazione Policlinico Universitario A. Gemelli IRCCS, Largo Francesco Vito 1, 00168 Rome, Italy; 5 Department of Health Science and Public Health, Università Cattolica del Sacro Cuore, Largo Francesco Vito 1, 00168 Rome, Italy; 6 Center for Global Health Research and Studies, Università Cattolica del Sacro Cuore, Largo Francesco Vito 1, 00168 Rome, Italy; 7 Gemelli Woman Health Center for Digital Health and Personalized Medicine, Università Cattolica del Sacro Cuore, Rome, Italy

**Keywords:** Gender medicine, Headaches, Migraines, Occupational health

## Abstract

**Introduction:**

Gender medicine is a new medical approach aimed at the study of the differences between women and men in terms of prevention, diagnosis, and the outcome of all diseases. Migraines are among these. They represent the most common neurological illness; they are most prevalent in adults between 20 and 50 years of age and are three to four times more frequent in woman than in men. Affecting people in working age, migraines are a problem that strongly impacts the psychophysical health and productivity of workers, regardless of the specific job task they have.

**Methods:**

A narrative review was performed, searching for the most relevant articles describing gender differences in people suffering from migraines, and particularly in workers.

**Results:**

Migraine global prevalence is 20.7% in women and 9.7% in men whereas prevalence in Italy is 32.9% for women and only 13.0% for men. This difference is partly explained by hormonal differences, as well as by differences in brain structure, genetic polymorphisms and neuronal pathways. Sex differences may also play a role in the progression from episodic to chronic migraine. In workers, migraines are mostly associated with strenuous physical work in men, whilst migraines triggered by night shifts, lack of sleep, or irregular sleep patterns are more common in women.

**Conclusions:**

To this day, the reasons of sex/gender disparity for migraine are still obscure. However, migraines, chronic migraine in particular, have a negative impact on the lives of all individuals affected by this disease, but particularly in women in which family cares and working activity are often superimposed. Migraine prevention strategies should be planned in workers through the occupational health physician.

## Introduction

Since 1998, the World Health Organization (WHO) has issued a “gender challenge” to nations and international organizations. It was a call for (i) a better appreciation of risk factors involving women’s health, (ii) the development of preventive strategies to lessen the impact of diseases that disproportionately plague older women (e.g., coronary heart disease, osteoporosis, and dementia), and (iii) an increased emphasis on understanding why men die sooner than women [1]. Stemming from this statement, a new field of scientific investigation was born: the so-called gender medicine (GM). In fact, in the last years, a large number of studies evaluated the differences between men and women in both health and disease that are common to both women and men. It has to be underlined that the meaning of terms “sex” and “gender” is different. Whilst “sex” refers to the set of biological characteristics with which a person is born (for example sex chromosomes, gonads, genitals, and sex hormones), “gender” refers to the socially defined characteristics that distinguish masculine from feminine, i.e., norms, roles, and relationships between individuals defined as men and women. GM involves the prevention, the diagnosis, the study of pathogenetic mechanisms, and the outcome of diseases, as well as the efficacy and safety of treatments. This reflects on biomedical studies: both experimental and clinical investigations should consider sex and gender as key variables and should provide answers to the plethora of questions raised by the discovery of a wide and relevant sex/gender disparity in human health and disease [2]. Migraine merges both sex and gender issues since both biological and sociocultural matters are of relevance in this disease.

## Gender differences in migraines

Migraines are among the most common neurological illnesses and many types of headaches, mainly chronic migraine, are responsible for important disabilities. Despite this, the importance of headaches is often undermined, misdiagnosed, and undertreated. Adults between 20 and 50 years of age are more often subjected to headaches, but children and adolescents also suffer from them. In adult women, migraine is three to four times more frequent than in men [3, 4]. Therefore, since the most affected people are in working age, migraines are a problem that strongly impacts the psychophysical health of workers, regardless of the specific job task they have [5].

The definition of migraines includes various conditions of varying intensity, frequency, and duration. It is therefore difficult to establish its prevalence [6]. The World Health Organization (WHO) along with the main international organization on headaches has been promoting a global campaign (*Lifting The Burden*, LTB) aimed to reduce the impact of headaches and obtain a “clear and objective perception of headaches burden”, translating prevalence and incidence data in disability. Globally, it is estimated that 46% of the adult population suffers from headaches, 42% of which are tension-type headaches (TTHs), whilst 11% are migraines [7]. The WHO classifies headaches, mainly chronic migraine, among disabilities and, reportedly, among the ten most incapacitating conditions for the general population, among the first five for women specifically [6].

The findings of the most relevant articles pertaining gender differences are summarized in Table 1.

**Table 1:** Main characteristics pertaining to gender differences of screened articles

Authors	Male/female incidence	Differences in duration, frequency and intensity of attacks	Estrogens hormonal influence	Hormonal, genetic and protein factors
Tonini MC, 2018 [3]	13% male–32.9% female	No significant differences for these parameters between women and men	Yes	Polymorphism of the ESR1* receptors
Vetvik KG and MacGregor EA, 2017 [4]	2 to 3 times more prevalent in women than in men	Women report longer duration of migraine attacks than men, whilst frequency and intensity are similar	Yes	CGRP**, Galanin, and Neuropeptide Y
Gupta S et al., 2011 [8]	2 to 3 times more prevalent in women than in men	These parameters are not considered in the article	Yes	CGRP**, TRPV1***, 5HT****
Krause DN et al., 2021 [9]	3 times more prevalent in women than in men	Frequency and severity of attacks varies during puberty, the menstrual cycle, pregnancy, the postpartum period and menopause	Yes	CGRP**
Allais G et al., 2020 [10]	18% male–43% female	Attack frequency is similar in men and women; attack frequency, intensity and duration are age-related and worst in women aged 30 and over	Yes	CGRP**
Labastida-Ramírez A et al., 2019 [11]	2 to 3 times more prevalent in women than in men	These parameters are not considered in the article	Yes	Functional interactions between ovarian steroid hormones, CGRP**, and the trigeminovascular system
Tsai C-K et al., 2022 [12]	2 to 3 times more prevalent in women than in men	Women have more frequent and intense headaches, and higher risk of chronicization	Yes	CGRP** and the trigeminovascular system

*estrogen receptor 1; **calcitonin-gene related peptide, ***transient vanilloid receptor 1, ****5-hydroxytryptamine

### Sex and gender interplay

In 2018, Tonini et al. [3] have underlined the important sex/gender differences in headaches, suggesting that migraine is very largely a female disease. The pathology follows a bimodal pattern for both genders, with two peaks at 35 years old and 50 years old, decreasing with age. In women, after puberty, migraine becomes three to four times more frequent than in men [3, 4]: the global prevalence is 20.7% in women and 9.7% in men [5]. The prevalence in Italy is 32.9% in women and only 13.0% in men [3]. As well as higher prevalence of migraine, women report longer attack duration, increased risk of headache recurrence, greater disability, and a longer period of time to recovery. The Migraine Disability Assessment questionnaire (MIDAS score) states that women are 1.34 times more likely than men to report MIDAS grade 4 (severe disability). This is a sex and gender difference since it is explained by hormonal effects, different reactions to pain, structural and functional differences in certain brain areas (the trigeminovascular system and the central nervous system’s pain), possible genetic factors but, also, by behavioral and nutritional habits. Estrogens play an important role in neuroexcitability, structure, and function of already mentioned brain areas, which could explain why women are more prone to migraine than men. In fact, the presence of sex hormone receptors in the trigeminovascular system seems to suggest that trigeminal neurons are sensitive to variations in the levels of these hormones [8] and it is well known that the incidence of migraine attacks in the course of female life is characteristic: the incidence increases rapidly during puberty, peaks during the reproductive years, and decreases after menopause [9, 13]. Hence, a critical role for sex hormones appears as conceivable. Furthermore, Allais and coworkers [10], in 2020, have also highlighted how several factors may modulate female predisposition to migraine throughout different factors including hormones, brain structure, genetic polymorphisms or mutations, life events, stress, and neuronal activity. An important role for hormones has also been highlighted in menstrual migraines: the premenstrual change in estrogens levels has been associated with migraine onset, whilst the same is not true for progesterone [14].

In the last years, the calcitonin gene–related peptide (CGRP) has been discovered to play a key role in migraine pathophysiology and its signaling could be a target of hormones that influence migraine [11]. Increased CGRP release from the trigeminal ganglia is a key component of a migraine attack. Antibodies against CGRP or the CGRP receptor are highly effective in treating migraine and the fact that they do not cross the blood–brain barrier suggests that therapeutic intervention within the peripheral trigeminal pathway is sufficient to abort or prevent migraine attacks. No sex differences in responses to CGRP antibodies were reported so far in the clinical trials, although such differences have been hypothesized [12]. These drugs are clearly effective in women but whether their actions are affected by hormonal status is not known [9, 15].

### Some gender-specific aspects

A major factor that causes lack in delivering and receiving optimal care, is stigmatization toward people that suffer from headaches. Because migraine is three times more likely to occur in women, this disease is still perceived as a “female illness” and, therefore, less legitimate [16]. The stigma has a negative impact on the lives of all individuals with migraine, but particularly in women, who are more commonly involved in traditionally uncompensated labor (e.g., housework and caregiving) compared to men. Social and environmental factors are also important in the pathogenesis of this disease. Victims of intimate partner violence and adverse childhood experiences have an increased risk for migraine. As women are predominantly the victims of these types of abuse, the relevance of gender in the etiology of this disease appears even more prominent [16].

## Migraines in workers: triggers and gender differences

As a general rule, the need to pay attention to the risk assessment and gender disparity at the workplace has so far been suggested [17]. Many factors that can positively or negatively influence the productivity and the perceived well-being of workers (perceived autonomy, social support, and job satisfaction) have been associated with work tasks and the workplace environment. In particular, factors that can trigger or exacerbate migraine attacks such as bright lights, especially in terminal workers, loud noises (especially if personal protective equipment (PPE) is not used properly, or at all), odors, smoke, solvent usage, and indoor air quality have been considered critical factors [18]. To be more specific, it is known how some air contaminants, such as volatile organic compounds (VOCs), aldehydes, and xylenes, are associated with headaches, both directly and through the onset of the so-called *Sick Building Syndrome* (getting worse the longer you are in a particular building and getting better after you leave*)*, of which one of the main symptoms, despite being non-specific, is the frequent occurrence of headaches [19, 20].

Relevant among workers are TTHs, associated to muscular discomfort of the neck and upper back; this type of headaches can be associated with wrong posture [21, 22]. Cigarán-Méndez et al., in 2019, highlighted how TTHs are associated to muscular pains differently based on sex: in women, muscle-skeletal pains appear to have a more prominent role in TTHs pathogenesis [23]. Despite therapeutical measures have been hypothesized through specific physiotherapy treatments [22], it is clear that to reduce the incidence of this type of headaches, a preventive strategy is essential, by adopting an ergonomically correct posture on the workplace through the usage of adequate sex-specific workstation (desk, chair, and so on) that should be personalized by the worker based on specific demands (profession type, but also height and weight, especially considering the sex difference in the incidence of TTHs associated with muscular pains). Furthermore, wrongful manual handling of loads, as well as being associated with lower back pains, has also been associated to TTHs, particularly in healthcare professionals [19].

Quantitative demands (high work load, long work hours, inflexible schedule) and emotional demands (stressful work atmosphere) negatively affect headache-related work and productivity [18, 24]. Work-related stress has been identified in numerous studies as a trigger in headaches, migraines, and TTHs [24, 25]. The role played by stress is evident, not only as a trigger in migraines, but also as a worsening factor; a similar role is recognized for sleep [26]. Circadian rhythm disruption has been described to possibly stimulate migraine attacks, highlighting shift work and night work as triggers. Shift work requires workers to be on duty during their biological resting phase and to be forced to sleep in their biological active phase. These aspects disturb sleep patterns and quality potentially increasing the risk of migraine onset. Since in Europe about 20% of the working population is involved in shift works, it appears evident the importance to understand shiftwork-related consequences on migraine [27, 28]. It has been highlighted how shift work and night work act as a migraine trigger and exacerbator more in women than in men [29]. Furthermore, it is not only night shifts, but also fixed evening work that is associated with migraine: evening workers have 56% increased odds of treatment-seeking migraine compared with fixed day workers and this difference is most pronounced among older employees [30].

Concerning healthcare workers, Kuo and colleagues have highlighted, in 2015, that headaches and migraines are more common in healthcare personnel, and particularly in nurses, as opposed to the general population [31]. In the last 2 years, during the COVID-19 pandemic, a higher incidence in headaches has been associated with PPEs [32]. Furthermore, it has been highlighted how the frequency and severity in headaches have increased in workers suffering from them before the pandemic, whilst the new onset of migraines has been highlighted in workers that were not affected before the pandemic. The severity and frequency of the episodes have been associated with prolonged usage of PPEs, constituting a higher risk in healthcare workers that adopted PPEs continuously and for many hours per day, especially for masks and FFP2 masks in particular [32, 33].

It has been highlighted in scientific literature how migraine education and management programs can increase productivity. Begasse and colleagues have reported an increase in productivity by 29–36% [18]. The role of prevention in reducing the impact of migraine in workers is evident, and it appears essential to plan strategies, through the occupational health physician, aimed at reducing the global burden of migraines, ensure the safety and health of workers and, on the other hand, to reduce the costs due to the headache-induced disability for both the national health systems and for productive activities.
